# P-1762. Assessing the Clinical Impact of Carbapenem Pre-authorization in a High ESBL Prevalence Setting: A Focus on the Middle East

**DOI:** 10.1093/ofid/ofae631.1925

**Published:** 2025-01-29

**Authors:** Amna Nuaman, Rania M El Lababidi, Wasim S El Nekidy, Emna Abidi

**Affiliations:** Cleveland Clinic Abu dhabi, abu dhabi, Abu Dhabi, United Arab Emirates; Cleveland Clinic Abu Dhabi, Abu Dhabi, Abu Dhabi, United Arab Emirates; Cleveland Clinic Abu Dhabi, Abu Dhabi, Abu Dhabi, United Arab Emirates; Cleveland Clinic Abu Dhabi, Abu Dhabi, Abu Dhabi, United Arab Emirates

## Abstract

**Background:**

Hospitals commonly employ preauthorization as a strategy to optimize carbapenems utilization. However, there is paucity of data regarding carbapenem restriction in environments with a high prevalence of extended-spectrum beta-lactamase (ESBL) organisms. This study aims to assess the impact of implementing a preauthorization policy for carbapenems on patient clinical outcomes and appropriateness of use.

**Figure:**
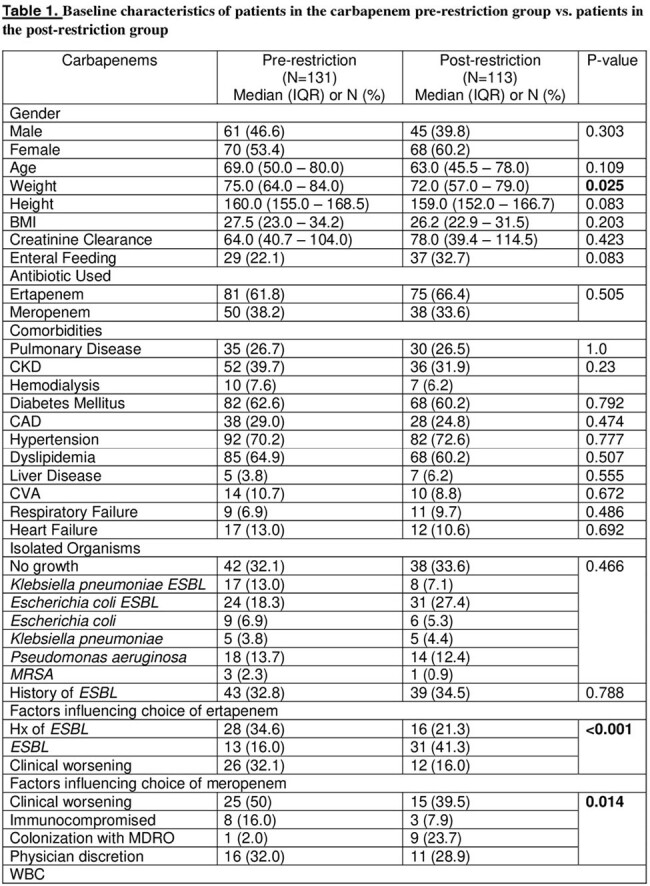

**Methods:**

An observational retrospective cohort study comparing data collected for patients from pre-restriction period between December 2021 and May 2022, and post-restriction period between June 2022 and December 2023 who were prescribed carbapenems including ertapenem or meropenem. The restriction policy was implemented in June 2022. We included adult patients ( > 18 years old) with active infections in acute care units (ACU).

**Figure:**
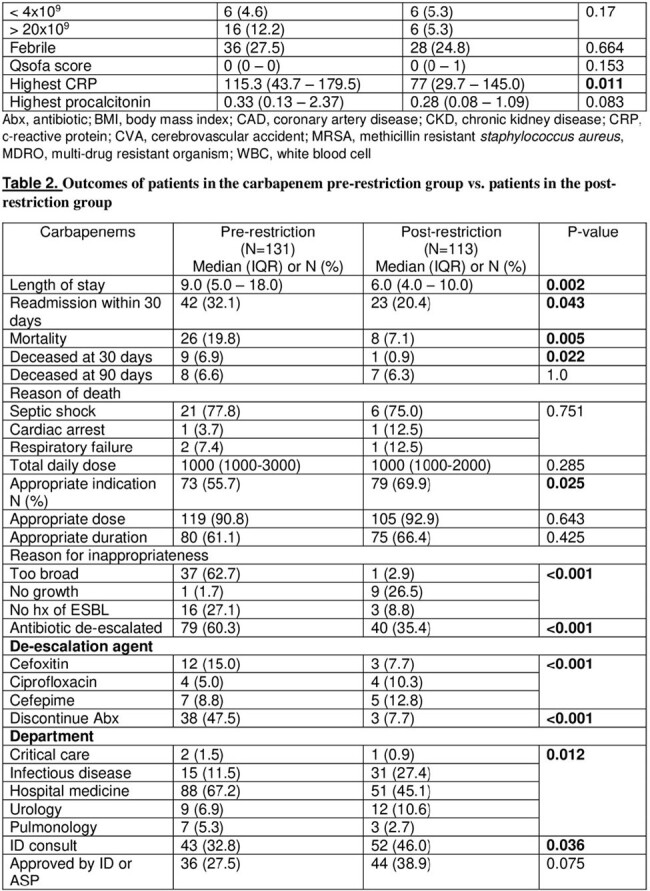

**Results:**

A total of 244 patient were included in the study with 131 in pre implementation period and 113 in post implementation period. The appropriate indication of carbapenems use was significantly higher in the post-restriction group 69.9% (79) vs the pre-restriction group 55.7% (73) (P=0.025). No significant difference was found between the two groups in terms of appropriateness of treatment duration (P=0.425). Antibiotic de-escalation was significantly higher in the pre-restriction group 60.3% (79) vs the post-restriction group 35.4% (40) (P< 0.001). The 30-day all cause mortality was significantly lower in the post-restriction group 7.1% (8) vs the pre-restriction group 19.8% (26) (P=0.005). Length of hospital stay (LOS) was significantly lower in the post-restriction group compared to the pre-restriction group; 6 vs 9 days (P=0.002). Furthermore, the 30-day readmission rate was 32.1% (42) of patients in the pre-restriction group vs 20.4% (23) in the post-restriction group (P=0.043).

Table 2

**Figure:**
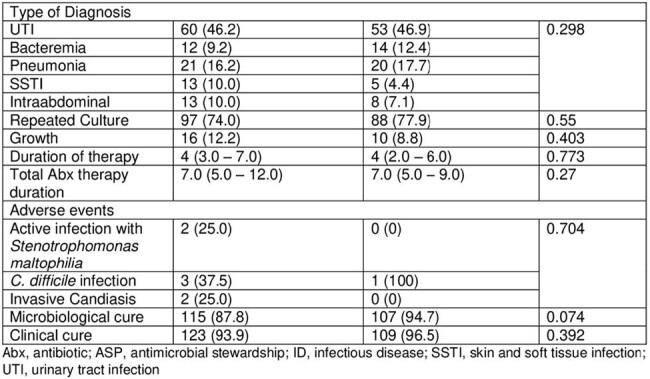

**Conclusion:**

Despite ESBL high prevalence, preauthorization policy for carbapenems prescription showed efficacy to optimize their use in terms of, both, appropriateness, and duration. Lower 30-day mortality, LOS and readmissions within ACU patients also confirmed better clinical outcomes. Larger studies are needed to corroborate these findings.

**Disclosures:**

**All Authors**: No reported disclosures

